# Dyspnoe – eine einjährige Leidensgeschichte

**DOI:** 10.1007/s00106-023-01313-x

**Published:** 2023-05-28

**Authors:** Claudia-Diana Pulbere, Stefan Edlinger, Georg Sprinzl

**Affiliations:** Klinische Abteilung für Hals-, Nasen-, Ohrenkrankheiten, Universitätsklinik St. Pölten, St. Pölten, Österreich

## Anamnese

Ein 6‑jähriger Junge wurde mit Überweisung von einem Fremdspital wegen seit einem Jahr rezidivierender Episoden von Dyspnoe und Stridor in unserer HNO-Notfallambulanz vorstellig. Die Mutter berichtete, es sei vor etwa einem Jahr zur versehentlichen Ingestion einer Glasscherbe gekommen, nachdem der kleine Patient in ein Trinkglas gebissen habe und dies zum Bruch des Trinkglases geführt habe. Akute Symptome fehlten zu Beginn komplett. Tage später litt der kleine B. unter einem intermittierend auftretenden inspiratorischen Stridor. Es erfolgten eine Reihe von Abklärungsuntersuchungen, wie z. B. eine Röntgenaufnahme des Thorax, ein Allergietest und mehrfache Auskultation. Alle Untersuchungen und die Bildgebung verliefen unauffällig. Der Junge erhielt ungefähr 9 Monate eine Dauertherapie mit einem inhalativen Kortikosteroid, auf weitere bildgebende Abklärungen wurde verzichtet. Knapp ein Jahr nach Symptombeginn kam es zu einer plötzlichen Verschlechterung, sodass die Rettung alarmiert wurde und der Junge ins örtliche Krankenhaus gebracht wurde. Dort veranlasste man eine Röntgenaufnahme von Hals, Thorax und Abdomen. Das Kind wurde in stabilem Allgemeinzustand per Nortarztwagen an die HNO-Abteilung des Universitätsklinikums (UK) St. Pölten transferiert.

## Befunde und Diagnostik

Auf eine HNO-ärztliche Untersuchung mit Inspektion der Hypopharynx- und Larynxebene wurde aufgrund des Kindesalters und der Evidenz der Befunde verzichtet. Eine Magnetresonanztomographie (MRT) konnte aufgrund des Alters und der Unruhe auch nicht stattfinden. Stattdessen wurde im stabilen Zustand ein seitliches Röntgenbild vom Hals angefertigt.

## Wie lautet Ihre Diagnose?

**Diagnose:** subglottischer Fremdkörper non recens

## Therapie und Verlauf

**Figure 1:**
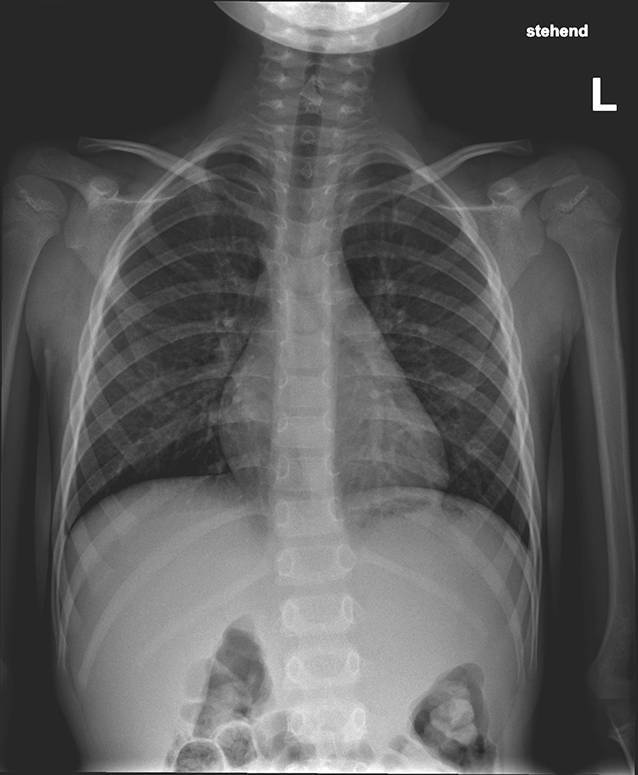

Die Bildgebung zeigte einen röntgendichten Fremdkörper in der Projektion der Halswirbelkörper 4/5, sodass die Lage des Fremdkörpers nun eindeutig bestimmbar war (Abb. 1 und 2). Es zeigte sich das anamnestisch genannte Glasstück, ca. 3 cm subglottisch liegend. Aufgrund des deutlichen Stridors wurde die Entscheidung zu einer sofortigen Entfernung gestellt. Ein translaryngealer Versuch war aufgrund von Verwachsungen frustran. Eine Entfernung per gezielter Tracheotomie war die Konsequenz. Die Entscheidung entfiel intraoperativ als akute Maßnahme. Nach Entfernung des Glasstücks (Abb. 3) wurde der Patient für 12 h orotracheal schutzintubiert, und das Tracheostoma konnte noch vor der Extubation verschlossen werden. Status des Kindes ein Jahr nach OP.: guter Allgemeinzustand, keine Beschwerden, Narbe bland (Abb. 4).

**Figure 2:**
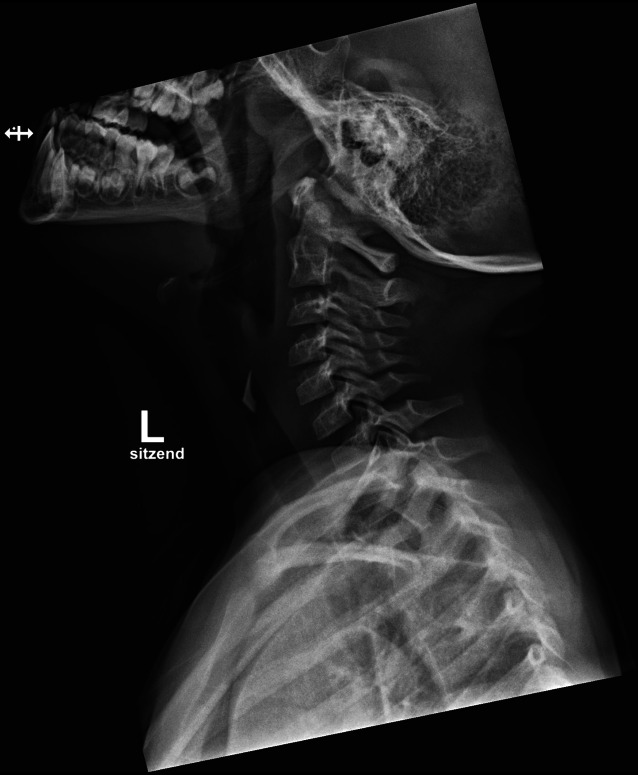

**Figure 3:**
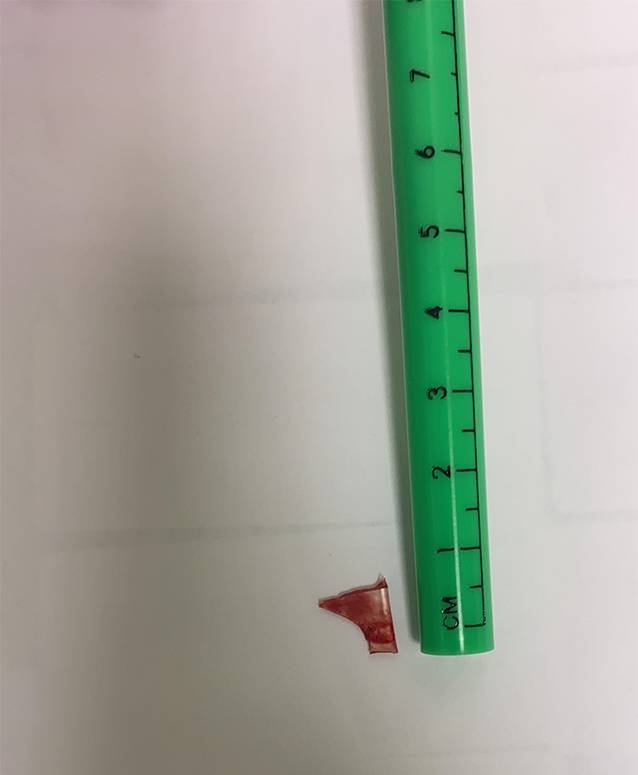

**Figure 4:**
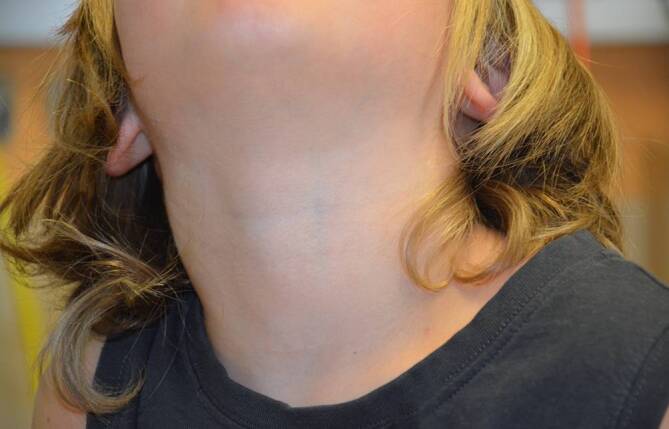

## Fazit für die Praxis

- Die asymptomatische Fremdkörperaspiration stellt eine seltene Entität im klinischen Alltag dar.
- Bei gegebener Anamnese sollte auch bei asymptomatischen Patienten eine rasche und zielgerichtete Abklärung erfolgen.
- Für Kinder ist es selbstverständlich, ihre Umgebung zu erkunden, indem sie Objekte in ihrer Umgebung sehen, berühren und schmecken.
- Leider kann ihre Tendenz, nichtessbare Gegenstände in den Mund zu nehmen, lebensbedrohlich sein.
- Nicht selten werden Fremdkörper dabei auch aspiriert.
- In der Regel präsentieren sich diese Kinder mit typischen und eindrucksvollen akuten Beschwerden.
- Diese können von Hustenreiz bis hin zu akuter Atemnot reichen.
- Aufgrund der selten vorkommenden, aber doch möglichen anfänglich fehlenden Symptomatik kann es zu Komplikationen und erschwerter Fremdkörperentfernung mit hohen intraoperativen Risiken kommen.

