# Erratum zu: Dyspnoe – eine einjährige Leidensgeschichte

**DOI:** 10.1007/s00106-023-01343-5

**Published:** 2023-07-20

**Authors:** Claudia-Diana Pulbere, Stefan Edlinger, Georg Sprinzl

**Affiliations:** Klinische Abteilung für Hals‑, Nasen‑, Ohrenkrankheiten, Universitätsklinik St. Pölten, St. Pölten, Österreich

**Erratum zu:**

**HNO 2023**

10.1007/s00106-023-01313-x

In diesem Artikel wurde die Bildunterschrift von Abb. 1, 2, 3 und 4 versehentlich vertauscht.

Die Abbildungen sollten wie folgt aussehen:

**Figure 1:**
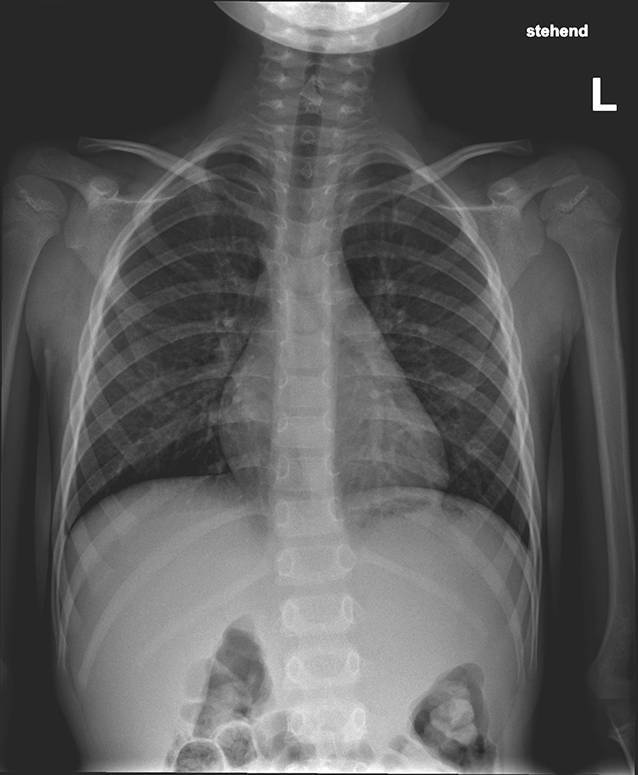

**Figure 2:**
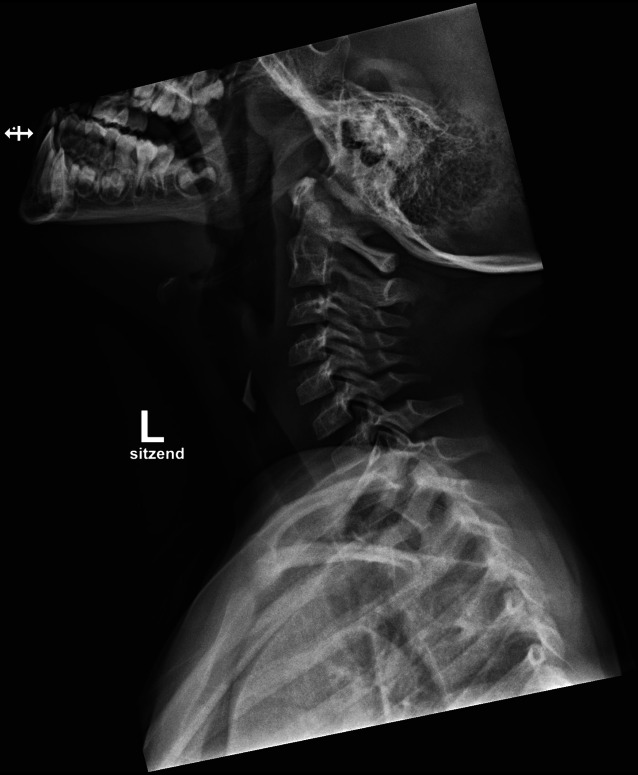

**Figure 3:**
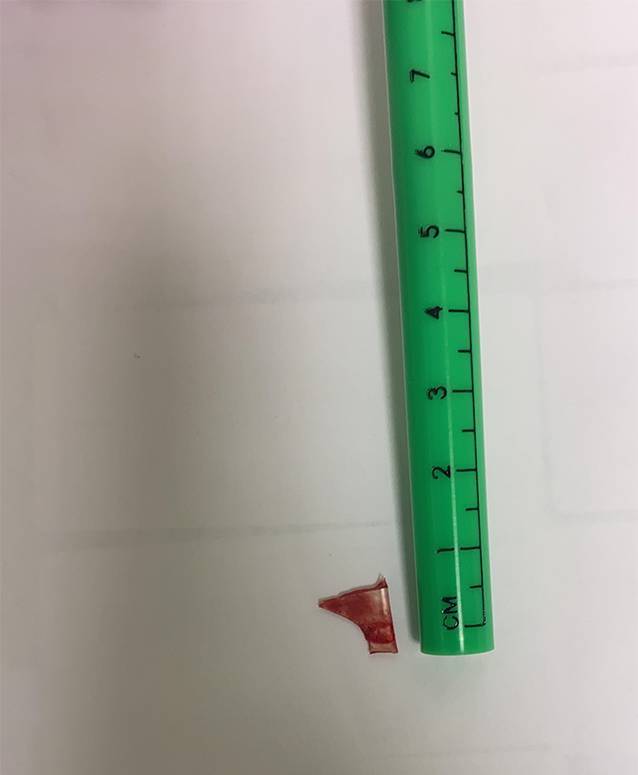

**Figure 4:**
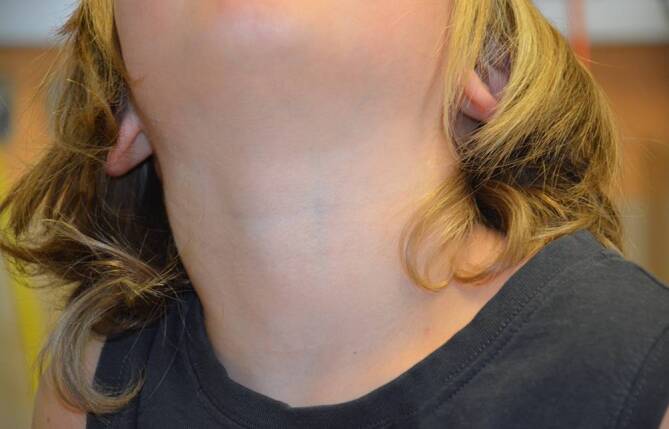

Der Originalbeitrag wurde korrigiert.

